# C-reactive protein and hemogram parameters for the nonsepsis SIRS and sepsis: what do they mean?

**DOI:** 10.1186/cc14131

**Published:** 2015-03-16

**Authors:** B Gucyetmez, HK Atalan, M Berktas, E Ozden, N Cakar

**Affiliations:** 1 International Hospital, Istanbul, Turkey; 2 Atasehir Memorial Hospital, Istanbul, Turkey; 3 Kappa Consulting, Istanbul, Turkey; 4 Antalya Memorial Hospital, Antalya, Turkey; 5 Acibadem University, Istanbul, Turkey

## Introduction

The aim of this study was to investigate the laboratory parameters as an indicator of sepsis. Sepsis is one of the most common reasons of mortality and morbidity in the ICU [1]. Thus, it is important to distinguish sepsis from nonsepsis SIRS. CRP and hemogram parameters may be fast, easy and affordable alternatives in distinguishing sepsis from nonsepsis SIRS. Eosinophil count (EoC), lymphocyte count (LymC) and neutrophil-lymphocyte count ratio (NLCR) are used as sepsis indicators [1,2].

## Methods

A total of 2,777 patients admitted to the ICU of two centers between 2006 and 2013 were evaluated retrospectively. The patients were diagnosed as SIRS(-), nonsepsis SIRS or sepsis at ICU admission by the consensus of two doctors in accordance with 1992 sepsis guidelines [3]. The patients who were under 18 years old, readmitted, immunosuppressive, SIRS(-) and whose laboratory values and outcomes were unknown were excluded. In total, 1,302 patients were divided into two groups as the nonsepsis SIRS group and the sepsis group. The patient's age, gender, diagnoses (medical, elective and urgent surgery), APACHE II, SOFA, CRP, WBC, neutrophil count (NeuC), LymC, NLCR, EoC, platelet, mean platelet volume, length of ICU stay and mortality were recorded by a third doctor. In the fully adjusted model, WBC, CRP, LymC, NeuC, NLCR and EoC were entered into the model.

## Results

A total of 1,302 patients were categorized as nonsepsis SIRS (816, 62.7%) and sepsis (486, 37.3%). In the sepsis group, age, APACHE II, SOFA, mortality, length of ICU stay, CRP, NLCR and EoC were higher; LymC was lower than in the nonsepsis SIRS group (*P *< 0.001 for each). Likelihood of sepsis (reference to nonsepsis SIRS) increased 2.62 (2.05 to 3.34), 2.02 (1.42 to 2.88) and 1.88 (1.36 to 2.60) times (OR (95% CI)) by the values of CRP >4.4 mg/dl, LmyC <500/mm^3 ^and NLCR >15.7 respectively in mutually adjusted multivariate logistic regression (*P *< 0.001 for each).

## Conclusion

CRP, LymC and NLCR may distinguish sepsis from nonsepsis SIRS. Thus, CRP and hemogram parameters may contribute to early diagnosis of sepsis.
